# Molecular Dynamics Reveal Key Steps in BAR-Related Membrane Remodeling

**DOI:** 10.3390/pathogens13100902

**Published:** 2024-10-15

**Authors:** Shenghan Song, Tongtong Li, Amy O. Stevens, Temair Shorty, Yi He

**Affiliations:** 1 Department of Chemistry & Chemical Biology, The University of New Mexico, Albuquerque, NM 87131, USA; 2 Translational Informatics Division, Department of Internal Medicine, The University of New Mexico, Albuquerque, NM 87131, USA

**Keywords:** pathogen–host interactions, endocytosis, membrane dynamics, BAR domain, helix kinks, molecular dynamics simulations, protein-lipid interactions

## Abstract

Endocytosis plays a complex role in pathogen-host interactions. It serves as a pathway for pathogens to enter the host cell and acts as a part of the immune defense mechanism. Endocytosis involves the formation of lipid membrane vesicles and the reshaping of the cell membrane, a task predominantly managed by proteins containing BAR (Bin1/Amphiphysin/yeast RVS167) domains. Insights into how BAR domains can remodel and reshape cell membranes provide crucial information on infections and can aid the development of treatment. Aiming at deciphering the roles of the BAR dimers in lipid membrane bending and remodeling, we conducted extensive all-atom molecular dynamics simulations and discovered that the presence of helix kinks divides the BAR monomer into two segments—the “arm segment” and the “core segment”—which exhibit distinct movement patterns. Contrary to the prior hypothesis of BAR domains working as a rigid scaffold, we found that it functions in an “Arms-Hands” mode. These findings enhance the understanding of endocytosis, potentially advancing research on pathogen-host interactions and aiding in the identification of new treatment strategies targeting BAR domains.

## 1. Introduction

Endocytosis plays a pivotal role in the pathogen–host relationship, serving both as a conduit for pathogen entry and as a crucial component of the host’s immune defense mechanisms [1,2]. Pathogens exploit endocytic pathways to invade host cells, facilitating their replication and spread. Conversely, the host utilizes endocytosis to internalize and degrade pathogens, activating immune responses. Understanding the molecular mechanisms of endocytosis is essential for deciphering how pathogens manipulate these processes and developing strategies to enhance host defenses, thereby improving infection control and treatment. Endocytosis and other essential intercellular transport functions such as exocytosis, phagocytosis, and pinocytosis rely on the dynamic bending and remodeling of the cell membrane [3,4,5,6,7,8]. Central to these processes is the formation of vesicular structures and microtubules, which are critical for cellular transport and communication. Therefore, an in-depth understanding of membrane dynamics is needed to further explore these bio-functional processes [9,10]. While the lipid membrane’s intrinsic fluidity and propensity for spontaneous curvature are fundamental, specialized proteins are required to overcome the membrane’s surface tension, induce sufficient curvature, and sustain that curvature [11,12]. The Bin1/Amphiphysin/Rvs167 (BAR) domain, which is a member of membrane-bending proteins, is a focal point in this context. The name BAR indicates its association with three prototypical proteins: mammalian Bin1, Amphiphysin, and yeast Rvs167 [13,14,15]. Proteins containing the BAR domain are predominantly involved in reshaping the cell membrane and dysfunctions in these proteins are linked to a range of pathologies including neurological disorders, developmental anomalies, and cancers [16]. Investigating the mechanisms of BAR domain-mediated membrane deformation extends beyond the basic and offers potential avenues for understanding presenting valuable opportunities to understand and potentially address a wide range of diseases. By elucidating the role of BAR domains in membrane dynamics, researchers can identify novel therapeutic targets and strategies that impact the pathogen–host relationship. This research not only deepens our knowledge but also paves the way for clinical applications, bridging the gap between molecular biology and medical treatment.

BAR domains perform their biological functions as dimers with a crescent shape. There are three major subfamilies of the extensive BAR superfamily: N-BAR and Classical BAR, F-BAR, and I-BAR [17,18,19,20]. Specifically, the N-BAR and Classical BAR subfamilies are characterized by shorter structural dimensions and distinctive positively charged amino acids distributed along their concave surfaces. The N-BAR domain can bind to the lipid membrane via these positively charged residues [21]. Additionally, the N-BAR dimer’s efficacy in membrane bending is further augmented by its N-terminal amphipathic helix, playing a critical role in the initial detection and location of membrane perturbations. In particular, Cui et al. posited a tripartite sensing–binding–bending mechanism [22]. Existing experimental results highlight that the rigid shape of N-BAR, the positively charged concave surface, and the N-terminal helices play pivotal roles in the sensing–binding–bending process, and affect the membrane-bending and remodeling ability of BAR-related proteins [23,24,25,26,27,28,29].

All-atom molecular dynamics (MDs) simulation is a powerful tool to study protein structure–function, by revealing protein complex activity at molecular and atomic resolutions [30,31,32,33,34,35,36,37,38]. Existing simulation studies on BAR domain proteins have explored the N-terminal amphipathic helix and some auxiliary domains that help activate, position, and fix BAR domains to membrane locations requiring curvature [17]. While simulations have been instrumental in exploring the roles of N-terminal amphipathic helices and auxiliary domains in BAR domain functionality, they likely do not play a decisive role in the actual remodeling of cell membrane curvature. Many successful molecular dynamics simulation studies have illustrated that BAR domains can bend the cell membrane even without the presence of auxiliary domains and N-terminal helices [39,40]. The positively charged residues on the concave surface of BAR dimers can tightly bind the BAR dimer to the negatively charged cell membrane. Further, losing the positively charged residues on the concave surface will literally erase the BAR dimer’s ability to bend the cell membrane [40]. These results suggest the binding process following a sensing–binding–bending mechanism. Current research is ambiguous in describing the process between the binding and bending stages of BAR-mediated membrane curvature. Some researchers believe that the BAR dimer plays the role of a rigid template or scaffold in “molding” the cell membrane [24,41]. It is undaeniable that the rigidity of the BAR itself is essential in the process of remodeling the cell membrane to a reasonable curvature. However, if the BAR dimer is only regarded as a rigid scaffold with a “molding” function, then the generation of curvature would depend hugely on the application of external forces, or the surface tension difference between the layers of membrane produced by the insertion of N-terminal helices. A series of simulations by Schulten et al. proved that BAR dimers can generate lipid membrane curvature under the absence of N-terminal helices, as long as the BAR dimers are properly placed on the surface of the membrane and maintain their shape [40,42]. These results suggest that the formation of curvature is attributed to fluidity and fluctuations in the membrane itself.

Our previous work revealed that, though PICK1 BAR domains demonstrate a certain level of rigidity, the presence of kinks introduces elasticity and flexibility rather than rigidity [43]. How BAR domains balance the rigidity and flexibility when performing their biological functions is of particular interest. Therefore, the goal of this paper is to use all-atom MD simulations to investigate the role of the BAR dimer in the membrane-bending and remodeling process with the presence of solution and ions in the biological environment.

## 2. Materials and Methods

### 2.1. Systems of MD Simulation

The initial structure of the BAR dimer used in the simulations is the crystal structure of the BAR domain of human Amphiphysin (PDB ID:4ATM) obtained from RCSB [44]. This is a classic N-BAR, same as the PICK1 BAR domains. The structure of the 4ATM BAR dimer contains residues 19-239 without N-terminal helices. Selecting only the BAR dimer eliminates the interference of the auxiliary domains and N-terminal helices in the simulations. To mimic the cellular environment, the MD simulations of the 4ATM BAR dimer were conducted in a system with explicit water molecules. The TIP3P water molecule was used to solvate the systems [45]. The CHARMM36m force field was used in all simulations [46,47]. The simulation system was generated by CHARMM-GUI [47,48] and visualized using visual MD (VMD) and Chimera [49,50]. A solvation box measuring 34 × 34 × 20 nm^3^ generated by CHARMM-GUI was used to house the 4ATM BAR dimer–membrane complex, ensuring ample room for any necessary deformations of the BAR dimer and membrane. The systems were neutralized with counter ions (K^+^ and CL^−^), and additional ions were added so that the ion concentration reached 0.15 M. According to previous simulations, the area where a single N-BAR achieves the best effect of bending and remodeling cell membranes is about 50–100 nm^2^ [39]. An oversized lipid membrane will not only hinder the generation of curvature but also consume unnecessary computing resources. Manually trimming the system (10.0 × 34.5 × 20.9 nm^3^ after trimming) is a two-pronged approach, as shown in Figure S1. The trimming process, involving reducing the number of water molecules, ions, and lipid molecules, was performed using the GROMACS2023.1 software package [51,52]. The number of trajectories, simulation timescales, and proportion of lipid molecules in the membrane are shown in Table 1 and Table 2. The composition and ratio of the types of lipid molecules in the membrane include DOPC, DOPE, DOPS, PI(4,5)P_2_, brain SM, and cholesterol, and the proportions are from Post et al., Matos et al., and Daum et al. [53,54,55]. Using more charged lipid molecules improves the ability of the 4ATM BAR to bind to the membrane.

All systems (trimmed) were energy-minimized to a maximum of 50,000 steps to remove non-physical contacts and interactions. Subsequently, NPT ensembles with 1,125,000 steps (6 stages of equilibrium simulations, 2250 ps in total) were executed to equilibrate the systems. The production simulations lasted 300 ns. The LINCS algorithm constrained bond lengths between heavy atoms and hydrogen atoms [56]. Simulations were conducted at a constant temperature of 310 K and the Nose–Hoover thermostat method was used [57], as reported in previous work, to replicate the cellular environment [58,59]. A constant pressure of 1 atm was maintained using the Perriello–Rahman method. Periodic boundary conditions were implemented in all three directions, and the Particle Mesh Ewald (PME) method was employed [60,61,62]. The cutoff distance of both the van der Waals interaction and Coulomb interaction was set to 12 Å.

### 2.2. Time-Resolved Force Distribution Analysis

The time-resolved force distribution analysis was conducted using the Time-resolved Force Distribution Analysis (TRFDA) software package [63], in conjunction with GROMACS2023. In TRFDA, residue-based pairwise forces for Coulomb and Van der Waals interactions were computed and observed over the process of the simulation. The interaction between residues was depicted by a pairwise force, which can be calculated using the sum of the atom-based pairwise forces. The details of the pairwise force calculations were described in a separate paper [64].

### 2.3. Interaction Area, Secondary Structure Analysis, and Helix Kink

The interaction area’s calculation and the secondary structure analysis of Chain B were completed using the GROMACS module [65]. Even though GROMACS does not have a direct module for computing the interaction area, the *gmx sas* [66] module was employed to calculate the solvent-accessible surface area, which can then be used to derive the interaction area by using Equation (1), as follows.

(1) $$S_{Interaction\;A-B}=\frac{{SASA}_{A}+{SASA}_{B}-{SASA}_{A\&B}}{2}$$

The secondary structure analysis of Chain B in the BAR domain was conducted using the *do_dssp* module in GROMACS [67]. The results of the analysis were subsequently processed and visualized using the GNUplot 5.2 software.

The Kink Finder 1.01 [68] software package was used to identify all helix kinks. The *Kink Finder* is a software package for identifying helix kinks based on their helical structure (twist angle of protein helix). More details on the Kink Finder can be found in the original paper [68].

## 3. Results

### 3.1. Structure Change in the BAR Dimer and Interaction of the BAR Dimer–Membrane

**Figure 1:**
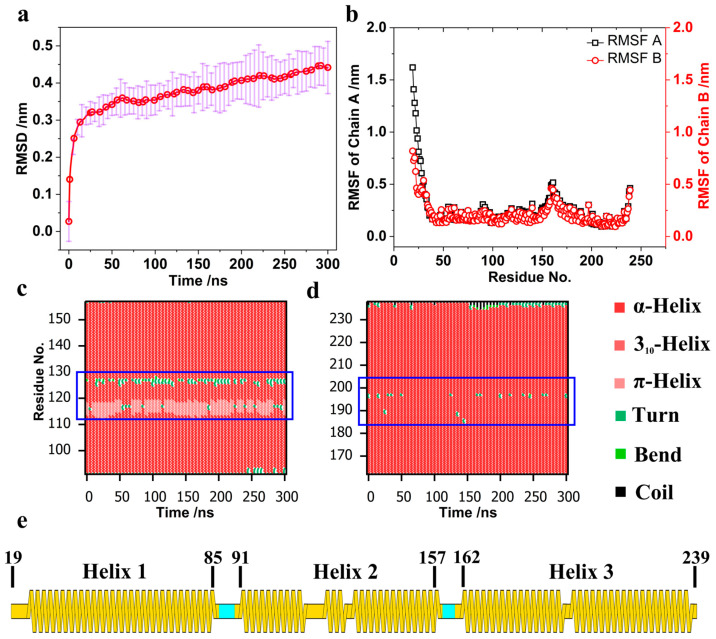
(**a**) Root Mean Square Deviation (RMSD) averaged over 10 trajectories. (**b**) Root Mean Square Fluctuation (RMSF). (**c**) Secondary structure analysis of Helix 2 in the 4ATM BAR monomer. (**d**) Secondary structure analysis of Helix 3 in the 4ATM BAR monomer. (**e**) Secondary structure segmentation of the 4ATM BAR monomer.

Following the completion of simulations, the Root Mean Square Deviation (RMSD) and Root Mean Square Fluctuation (RMSF) were computed to probe the comprehensive structural changes in the 4ATM BAR dimer during the membrane-bending process. As shown in Figure 1a,b, the RMSD exhibits a rapid increase within the first 25 ns, indicating that the 4ATM BAR dimer quickly transitions from its crystal structure in solution to a “functional configuration” on the membrane. Over the subsequent 275 ns, the RMSD increases gradually, yet remains relatively low, suggesting minimal structural changes in the BAR dimer during its interaction with the lipid membranes.

To further investigate structural fluctuations, RMSF analysis was performed, revealing residues with significant mobility. Chains A and B, which share identical sequences, exhibit similar RMSF patterns and secondary structure profiles. Notably, residues 85–91 and 157–162, which connect helices and possess a coil secondary structure, show an elevated RMSF (Figure 1e). Additionally, the N-terminus (residues 19–40) and the central regions of Helix 2 and Helix 3 (residues 110–130 and 185–200) display a higher RMSF than other helical regions. For most Amphiphysin BAR dimers [18], such as PICK1 [38], Helix 1 is shorter than other helices. The main function of Helix 1 is to stabilize the monomer by forming a coiled-coil structure with the other two helices [69] and to connect the N-terminal helix with the BAR dimer. Helix 1 also has a smaller curvature relative to the other helices; thus, structural analyses of BAR monomers typically focus on Helix 2 and Helix 3. However, in the 4ATM BAR, Helix 1 is longer than in PICK1, rendering the N-terminus of Helix 1 more dynamic. This increased activity suggests that Helix 1’s N-terminus participates in membrane binding. This point will be further examined in our analysis of hydrogen bonding interactions between the BAR domain and the membrane. Secondary structure analysis of the 4ATM BAR monomer, shown in Figure 1c,d, aligns with RMSF findings, with regions of residues on Helix 2 and Helix 3 (residues 110–130 and 185–200) displaying increased secondary structure shifts. Based on our previous work, these regions correspond to kink areas in Helix 2 and Helix 3 of the 4ATM BAR [43]. Using the Kink Finder software, we identified kink regions in Helix 2 (residues 108–120 and 122–134, centered on VAL114 and PHE128) and Helix 3 (residues 188–200, centered on LEU194). These results are consistent with our secondary structure analysis, showing that residues in the kink regions exhibit greater activity than other regions in Helix 2 and Helix 3. The presence of kinks introduces functional diversity to the helices, particularly in transmembrane proteins, despite their predominantly helical structures [70]. However, the specific impact of these kinks on BAR domain-mediated membrane curvature generation remains poorly understood, warranting further investigation.

**Figure 2:**
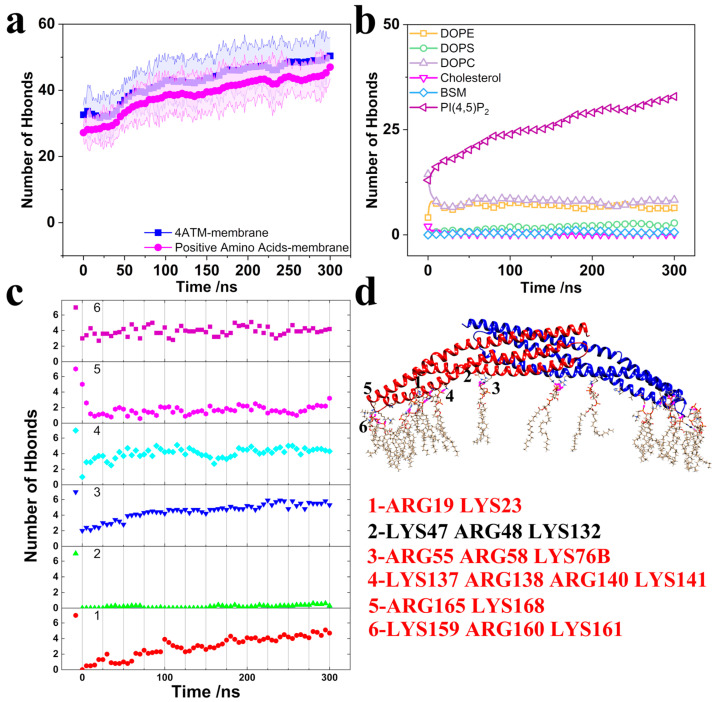
Hydrogen bonds. (**a**) Number of hydrogen bonds with error bands. Blue lines represent hydrogen bonds between the 4ATM BAR domain and the membrane; pink lines represent hydrogen bonds between positively charged amino acids and the membrane. Solid lines indicate the mean number of hydrogen bonds, while faded lines depict error bands. (**b**) Hydrogen bonds between lipid molecules and the membrane. (**c**) Hydrogen bonds between amino acid residue groups and the membrane. (**d**) Positions of amino acid residue groups and bound lipid molecules. Residues colored red have hydrogen bond interactions with lipids; residues colored black have minimal hydrogen bonding with lipids.

To elucidate the interactions between the 4ATM BAR dimer and the membrane, we calculated the number of hydrogen bonds using the GROMACS2023 software package [71]. Figure 2a presents the number of hydrogen bonds formed between the positively charged amino acids on the concave surface (LYS, ARG, and HIS) and the membrane, as well as the total number of hydrogen bonds between the entire 4ATM BAR dimer and the membrane. Consistent with previous studies, the positively charged amino acids on the BAR dimer’s concave surface play a critical role in mediating its binding to the membrane [72,73,74]. Specifically, the BAR dimer primarily interacts with the negatively charged PI(4,5)P2 in the lipid membrane, aligning with the experimental findings of Daum et al. [55] (Figure 2b). As illustrated in Figure 2d, each monomer within the BAR dimer possesses approximately six clusters of positively charged residues distributed along the concave surface. We calculated the number of hydrogen bonds between each of these clusters and the membrane (Figure 2c). Except for group 2 (LYS47, ARG48, and LYS132), each cluster forms around four hydrogen bonds with the membrane. Collectively, these positively charged residues across the dimer contribute to the formation of approximately 40 hydrogen bonds, consistent with the overall data shown in Figure 2a for the positively charged amino acids. These ten clusters of positively charged residues (five groups in BAR domain and two domains in 4ATM BAR dimer) are pivotal in facilitating the binding of the 4ATM BAR dimer to the membrane, making them potential targets for BAR protein engineering. Modifying the number or positioning of these key residues on the concave surface could significantly alter the binding affinity of the 4ATM BAR dimer to the membrane. Notably, the residues in group 1 (ARG19, LYS23) originate from the N-terminus of Helix 1 in the 4ATM BAR monomer. During membrane binding, the N-terminus of Helix 1 bends downward, correlating with the elevated RMSF observed at the N-terminus of the 4ATM BAR monomer. The extended Helix 1 in the 4ATM BAR monomer enhances the stability of the BAR dimer’s interaction with lipid membranes, thereby improving its efficacy in membrane bending.

In the previous analysis, we identified key residues in BAR dimers, namely positively charged residues on concave surfaces and helix kinks. Another group of residues that influence structure stability are major force-bearing residues between helices within the monomers. The BAR monomer is composed of three long helices. Those helices form a coiled-coil-like structure to achieve mutual stability [69]. In coiled-coil structures, the main interactions holding the helices together are hydrophobic interactions and electrostatic interactions between charged residues. The Time-resolved Force Distribution Analysis (TRFDA) software package was used to calculate interactions between helices and identify the major force-responsive residues. The residue-based punctual stress (visualization of FDA) and diagrams locating the residues are shown in Figures S2–S4. The top ten residues with the highest force between helices are shown in Table 3. According to the ranking of force-bearing residues in Table 3, the top two residues are both charged amino acids. There are more charged amino acids in the list, especially when extending the list to the top twenty residues. These charged residues can interact through electrostatic forces, serving as anchor points akin to those in the coiled-coil structure [75]. Besides the charged residues, we identified several phenylalanines (PHEs), tyrosines (TYRs), and tryptophans (TRPs) among the ranking. These amino acids possess large hydrophobic side chains with benzene rings, allowing them to form a hydrophobic core between helices [76]. The electrostatic interaction anchor points and hydrophobic cores are distributed between the helices, ensuring the stability of their relative positions and, consequently, the stability of the monomer structure [77]. By altering the number and position of these major force-bearing residues, the stability of the monomer can be modified. This provides a strategic approach for enhancing or tuning protein function [78].

### 3.2. Brief Analysis of the Bending Mechanism of Membrane Curvature

During the process of curvature generation, the 4ATM BAR domain interacted with the membrane and underwent a conformational change. Traditionally, the role of BAR domains in membrane bending has been conceptualized as that of a rigid scaffold, with membrane curvature attributed to surface tension differences induced by the insertion of N-terminal amphipathic helices and the fluctuation in lipid molecules. However, in our simulations the 4ATM BAR dimer configuration lacked N-terminal helices. The fact that significant curvature was generated in the absence of these helices prompts us to explore alternative mechanisms beyond the rigid scaffold hypothesis, particularly focusing on the influence of helix kinks on membrane curvature generation.

**Figure 3:**
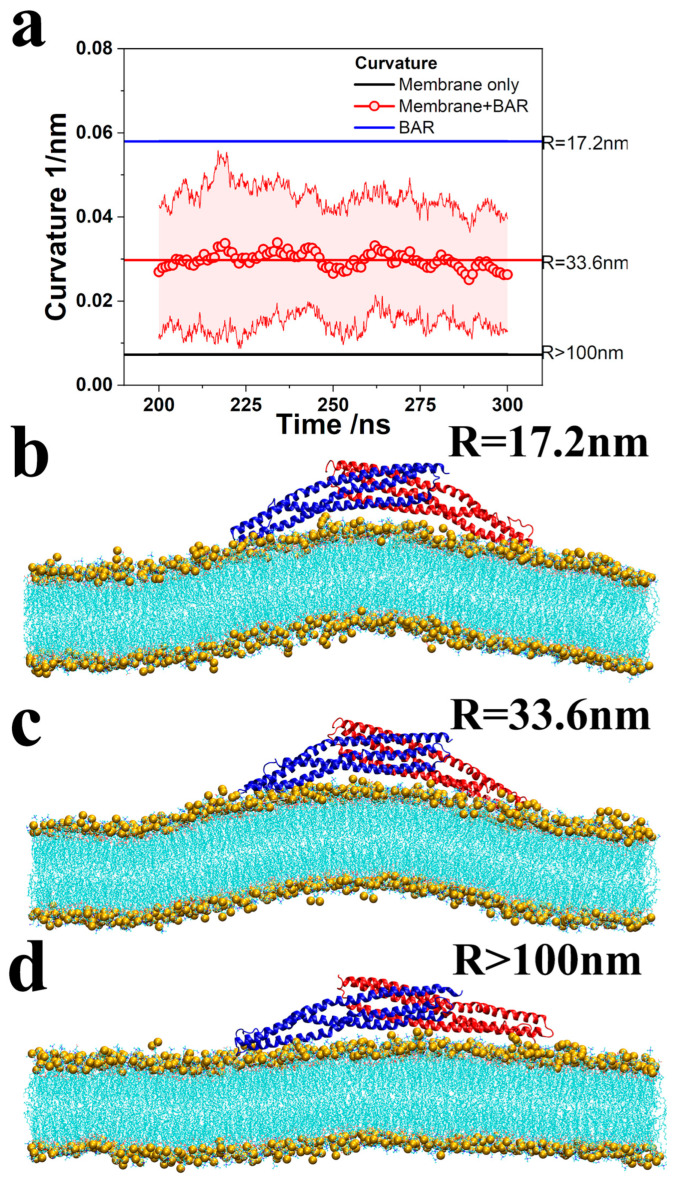
Curvature of the membrane and 4ATM BAR dimer. (**a**). Red line and dots with error band: Curvature of the membrane with the 4ATM BAR over time. The results are the average of 10 trajectories. Black line: Curvature of the membrane without the 4ATM BAR. The curvature is the average of 300 ns simulations. Blue line: Curvature of the 4ATM BAR crystal for RCSB. (**b**–**d**): Curved membrane with the 4ATM BAR showing the fitting radiuses. Lipid molecules are shown in cyan, in line style, while phosphorus atoms are shown in yellow.

To investigate this, we calculated membrane curvature across 10 simulation trajectories. Lipid membrane curvature was determined using a least squares circle fitting algorithm, with the phosphorus atoms of the lipid molecules beneath the 4ATM BAR dimer serving as reference points for the curvature calculations. The curvature, obtained by fitting the phosphorus atoms within a range of 160 × 10 Å^2^, was averaged and is presented in Figure 3a for the final 100 ns of the simulations. For comparison, Figure 3a also includes the calculated membrane curvature in the absence of the 4ATM BAR dimer and the curvature induced by the 4ATM BAR dimer itself.

Our simulations confirm that the 4ATM BAR dimer binds to lipid membranes and induces curvature even in the absence of an N-terminal helix. The average curvature of the lipid membrane during the last 100 ns of the 10 simulation trajectories was 0.0297 nm^−1^, corresponding to a fitting circle radius of 33.5 nm. In contrast, simulations without the 4ATM BAR dimer showed that the lipid membrane spontaneously developed curvature due to molecular fluctuations and fluidity, but this curvature was minimal, with an average value of only 0.0074 nm^−1^ (fitting circle radius ≈ 130 nm). A comparison of the lipid membranes in Figure 3c,d clearly demonstrates that the presence of the 4ATM BAR dimer induces significant membrane bending. These findings imply that the 4ATM BAR dimer plays a critical role in generating lipid membrane curvature, as membrane fluidity and fluctuation alone are insufficient to produce substantial curvature in the absence of the BAR dimer. Additionally, the curvature of the 4ATM BAR dimer concave surface was calculated as 0.0581 nm^−1^ (radius of the fitting circle, R = 17.2 nm, as shown in Figure 3b). Throughout most of the simulation, the membrane curvature was notably smaller than the curvature of the BAR dimer’s concave surface. The inability of the lipid membrane’s curved and convex regions to fully conform to the dimer’s concave surface suggests that the 4ATM BAR dimer does not function as a rigid scaffold in mediating membrane curvature. If it did, we would expect the lipid molecules to align more closely with the BAR dimer, resulting in membrane curvature similar to that of the dimer’s concave surface. Therefore, we conclude that the 4ATM BAR dimer is not merely a rigid scaffold in the membrane-bending process, and the key factor in generating membrane curvature is not the fluidity and fluctuation in lipid molecules alone.

It must be acknowledged that the curvature of the lipid membrane in the simulations was not stable, as shown by the error bands in Figure 3a. Although the average curvature obtained is very close to that obtained in the previous work on N-BARs by Yin et al. (~34 nm) [39], the standard deviation of our simulation is about 0.015 nm^−1^. After analyzing the simulation system settings and molecular dynamics characteristics, we believe the reasons for the unstable curvature may be as follows. First, the size of the membrane used in the simulation is about 10.0 × 34.5 nm^2^ (345 nm^2^), which is larger than the area that a single N-BAR can handle. According to estimates by Yin et al. [39], such a membrane requires 3–6 N-BARs to achieve optimal bending curvature. In this regard, we selected the lipid molecules directly below the BAR when calculating the curvature to reduce the influence of this factor. Second, the 4ATM BAR dimer is combined with the lipid membrane through hydrogen bonding interactions. In the H-bond analysis of Yu et al. [40], the hydrogen bonds between the BAR dimer and the lipid membrane were constantly switching. In other words, the binding between the BAR dimer and the lipid membrane is dynamic. The advantage of this is that the binding configuration between the BAR dimer and the lipid membrane can be adjusted in a time-saving and labor-saving manner. The BAR dimer that completes the biological function can also be easily detached. Lastly, curvature instability may result from the limitations in computing resources required by the MD simulation method. Given the limitations, the biological process of dozens or even hundreds of BAR dimers jointly remodeling lipid membranes into vesicles or microtubules can hardly be reproduced computationally. Therefore, current simulation software generally uses periodic boundary conditions to simulate systems with infinite size. This leads to the fact that when the lipid membrane in the simulation box bends, the equivalent configurations at both ends are not infinitely continuous lipid membranes, and cracks are introduced. Surface tension and hydrophobic interactions between lipid molecules both create a tendency for the membrane to return to flat. As a result, the membrane curvature fluctuates. Performing multiple simulations and averaging the curvature smooths out this deviation, reflecting the universal curvature. Given that the average curvature of lipid membranes is very close to the previous simulation results of Yin et al. [39] and Arkhipov et al. [42], we believe that our simulation results are reliable. It is reasonable to use our simulations to consider the role of the 4ATM BAR dimer in bending lipid membranes other than a rigid template.

**Figure 4:**
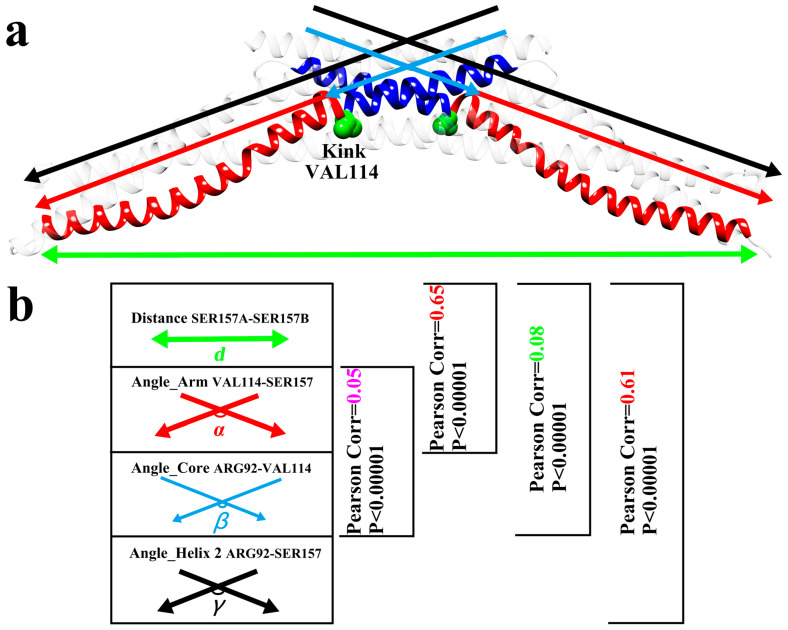
Pearson correlation. (**a**). Segmentation of the 4ATM BAR. Red: “arm segments”. Blue: “core segments”. Black: Helix 2. Green: Span of the 4ATM BAR dimer. Distance between SER157 Chain A and SER157 Chain B. (**b**). Pearson correlation between the (***d***) distance and angles (***α*, *β*, and *γ***) and Pearson correlation between the “arm segment” (***α***) and “core segment” (***β***).

Since we have questioned the theory that the BAR dimer is a rigid scaffold, we should explore the role that the 4ATM BAR dimer plays in the process of bending the lipid membrane. It is known that the presence of helix kinks will break the integrity of the helix, leading to the splitting of the helix into two parts, adding extra structural diversity to the helices [68,70]. The central residue (VAL114) of the main kink region of Helix 2 was selected as the segmentation point. Helix 2 in both monomers was divided into two parts: the “core segment”, ARG92-VAL114, and the “arm segment”, VAL114-SER157. As shown in Figure 4a, four parameters were defined, which are the angles between the two parts of the Helix 2 in the dimer (***γ***), the angle between the two “core segments” (***β***), the angle between the two “arm segments” (***α***), and the span of the dimer (***d***). The above four parameters in ten trajectories were calculated, and the Pearson correlations between each of the individual three angles (***α*, *β*, and *γ***) and the span (***d***) were calculated. The results are shown in Figure 4b. The calculated values of the four parameters (***α*, *β*, *γ*, and *d***) for the 10 trajectories are shown in Figure S5. The Pearson correlation coefficient between ***α*** and ***d*** is 0.65, showing a strong positive correlation. The Pearson correlation coefficient between ***β*** and ***d*** is 0.08 and the Pearson correlation coefficient between ***α*** and ***β*** is 0.05, which are both close to no correlation. From this, two conclusions can be inferred. First, the helix kinks in the 4ATM BAR dimer divided the helix into multiple distinct segments, providing additional structural diversity to the helix just as helix kinks in transmembrane proteins do [68,70]. Second, in the process of curvature generation, the span of the BAR dimer will fluctuate to a certain extent, rather than remain constant, acting as a rigid scaffold. The oscillation of the “arm segment” of the monomers causes span fluctuation rather than a change in the angle between the two monomers. This is also demonstrated by the Pearson correlation coefficient between ***γ*** and ***d***. Although the Pearson correlations between ***α***–***d*** and ***γ***–***d*** are both strong positive correlations, the Pearson correlation coefficient between ***α*** and ***d*** is slightly larger than the correlation coefficient between ***γ*** and ***d***. Since ***γ*** is the angle between Helix 2 in both monomers, including the “core segment”, the correlation between ***γ*** and ***d*** is weakened. In our previous work, the interaction area was used to describe the change in the opening angle between monomers [43]. For the simulation of the 4ATM BAR, the interaction area was also calculated, as shown in Figure S6. According to the interaction area in Figure S6, the opening angle between the 4ATM BAR monomers slightly oscillated but was mostly unchanged. Due to the peculiarities of the structure, the interaction area is highly related to the angle between “core segments” (***β***). The stability of the interaction area also indicates a tendency for “core segments” to move uncorrelated with other segments. When the 4ATM BAR dimer attempts to bend the lipid membrane, the “arm segments” of the two monomers will constantly swing around kinks. We believe that this oscillation plays an irreplaceable role in generating the curvature of lipid membranes. The two “arm segments” of the 4ATM BAR dimer act like two strong arms, squeezing the lipid molecules toward the middle to create curvature in the membrane. To assess this hypothesis, further analysis was performed.

**Figure 5:**
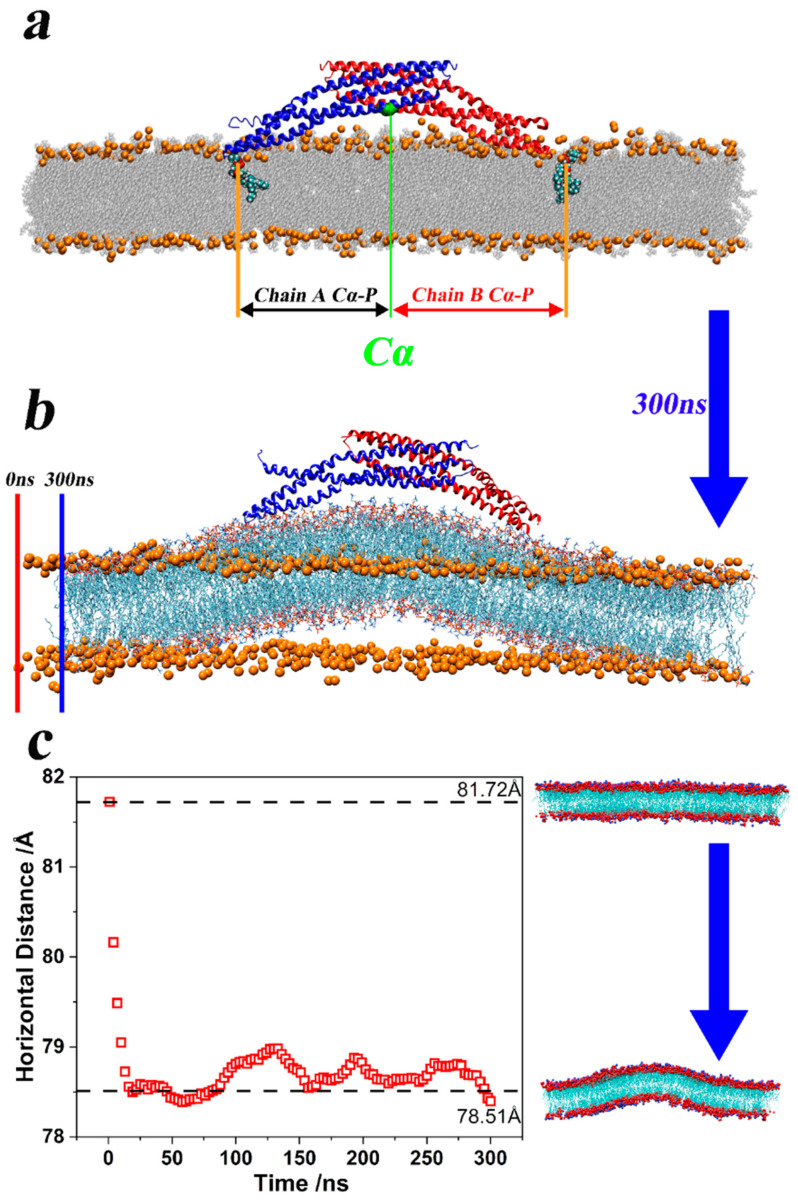
Centripetal extrusion and upward bulge of the lipid membrane. (**a**). Definition of the horizontal distance from the lipid molecules bound at both ends to the central residue. The central residues are GLY69A and GLY69B, and the reference atom is Ca. The reference atom of lipid molecules is the phosphorus atom. (**b**). The 4ATM BAR with the membrane after 300ns of simulation. The orange atom marks the position of the membrane at 0 ns. (**c**). The average horizontal distance between lipid molecules and the center of the concave surface of the 4ATM BAR dimer. The calculated result is the average of ten trajectories. When calculating, the reference atom on the lipid molecule is a phosphorus atom. The average coordinates of GLY69A and GLY69B determine the center of the concave surface of the 4ATM BAR dimer.

To prove that the two “arm segments” of the 4ATM BAR dimer “squeeze the membrane” as aforementioned, we calculated the horizontal distances between representative lipid molecules around two ends of the 4ATM BAR dimer and central residues (GLY69A and GLY69B). The definition of distance is shown in Figure 5a. In the initial configuration of production simulations, these lipid molecules were combined with residues near the endpoints of the 4ATM BAR dimer through hydrogen bonding interactions. The average horizontal distance between lipid molecules and the center of the concave surface of the 4ATM BAR dimer (GLY69A and GLY69B) was calculated and demonstrated in Figure 5c. As the simulation proceeded, the horizontal distances first decreased rapidly, and then showed a slightly oscillating downward trend. Figure 5b also demonstrates the horizontal distance decreasing in the membrane. The orange atoms in Figure 5b were the positions of the phosphorus atoms of the lipid molecules at the 0 ns moment in the representative trajectory. At this time, the lipid membrane was in a plain state. After 300 ns of simulation, the lipid molecules were illustrated in line style (colored in red for Oxygen and cyan for Carbon). At this point in the simulation, the lipid membrane exhibited a pronounced upward bulge, and its horizontal length was shorter than before the simulation. If the 4ATM BAR dimer acted solely as a rigid scaffold, the membrane would not experience horizontal compression, and lipid molecules would move only in the vertical direction. However, since the membrane is a continuous material, any upward movement of the lipid molecules must be accompanied by lateral compression or displacement. This behavior suggests that the 4ATM BAR dimer’s bending effect on the lipid membrane likely originates from interactions with lipid molecules bound to the dimer’s endpoints. The endpoints of the dimer first contact and then bind to the lipid molecules on the membrane. Then, through the oscillating swing of the BAR dimer “arm segments” around the helix kinks, the lipid molecules were continuously squeezed toward the center of the concave surface. Then, the number of lipid molecules in the center of the concave surface increased and the lipid membrane bulged upward. The bulged lipid molecules combined with the positively charged residues in the middle of the concave surface of the 4ATM BAR dimer to form a curved membrane structure. In this process, the 4ATM BAR dimer acted like two strong arms, squeezing the lipid molecules within their reach, and generating an upward bulge. The convex lipid membrane was then bound and fixed by the positively charged amino acids on the concave surface of the dimer to form a relatively stable curvature. Due to the limited space in the simulation box, when some lipid molecules move toward the center, other lipid molecules may also be squeezed away from the center. Through visual selection, several representative lipid molecules were chosen and the distances from selected lipid molecules to GLY69A and GLY69B were calculated. The selected lipid molecules are all lipid molecules that bind to one end of the BAR dimer at the initial stage of the simulation. As the “arm segments” of the 4ATM BAR dimer swing, these lipid molecules continue to move toward the center of the concave surface. This indicates that during the membrane-bending and remodeling process, the BAR dimer’s “arm segments” can “grab” and “gather” lipid molecules, essentially functioning as hands and arms.

## 4. Discussion

Past research has proposed a sensing–binding–bending mechanism of the BAR protein complex. The focal point here lies in further exploration of the role of BAR dimers in the “bending” process. By introducing helix kinks as the dividing points, the 4ATM BAR monomers were divided into “core segments” and “arm segments”. Our study provides evidence that the swing of the “arm segments” drives contraction and relaxation of the 4ATM BAR dimer and, conversely, the “core segments” are responsible for the enhancing of membrane curvature. The positively charged amino acids at both ends of the 4ATM BAR dimer contact and grasp the lipid membrane. The “arm segments” of the 4ATM BAR dimer then swing around the helix kinks. The lipid membrane is continuously squeezed centripetally by the swing of the 4ATM BAR dimer, causing the lipid membrane to bulge upward. Eventually, the upward protrusions will be bound by the positively charged amino acids on the concave surface of the 4ATM BAR dimer, forming a stable curvature of the lipid membrane. Past research has identified the helix kinks in BAR dimers, but their study of kinks was limited to the curvature of the BAR caused by kinks [74]. We found that kinks act as perturbation points for helices in monomers of the BAR domain, which adds extra structural diversity to the BAR dimer. However, this diversity is obstructive to rigid scaffold function and should be improved. A large number of bioinformatics studies on BAR domains showed that the helix kinks of BAR dimers are conserved at least among elements of the same subfamily with a similar morphology [18,79]. Given the differences in the horizontal length of the lipid membrane calculated before and after simulation, we question the assumption that the BAR dimer serves as a rigid template. Looking beyond the rigid scaffold hypothesis, we explored the role of the BAR dimer in the bending process from another angle. Specifically, we looked at how the BAR dimer’s motion can be modeled by a pair of “arms and hands” which grab and squeeze the membrane inward.

It is important to acknowledge the following limitations inherent in our methodology: all-atom MD simulations cannot simulate full-scale annular lipid membranes, and the computational resource limits the simulation time length and the system size. Despite successfully calculating the average horizontal distance between lipid molecules and the center of the 4ATM BAR dimer’s concave surface, it is difficult for us to track the movement trends of all lipid molecules during the simulation process. Lastly, we only selected representative molecules for calculation and display. Clarifying detailed mechanisms of BAR dimer bending and lipid membrane remodeling is crucial for advancing our understanding. Future investigations should elucidate the protein engineering and practical application of BAR dimers in biopharmaceutical and other fields.

## 5. Conclusions

In this article, we performed a series of simulations on the 4ATM BAR dimer. In past simulations, we wanted to study the structural changes during the interaction of the BAR dimer with the membrane. To do this, external forces were applied to a monomer of the PICK1 BAR dimer, both in upward and downward directions [43]. Although we have speculated on the structural changes and key residues of the BAR dimer, there is a lack of clear understanding of the degree of structural changes in BAR domains when they actually exert biological functions. This motivated us to conduct MD simulations of the interaction between the BAR dimer and membrane. Based on the simulations, three types of key residues on the 4ATM BAR dimer were identified: positively charged residues on the concave surface, kinks that affect the curvature and flexibility of the BAR dimer, and force-bearing residues that stably bind the three helices in the monomer together. Further, we found that helix kinks divided the dimer into “core segments” and “arm segments”, and each segment exhibits a different movement trend. This finding conflicts with the previous hypothesis that the BAR dimer functions as a rigid scaffold. As the 4ATM BAR dimer interacts with the lipid membrane, the “arm segments” continue to swing, which changes the span of the 4ATM BAR dimer. The swing of the “arm segments” will inevitably squeeze the lipid molecules in the membrane, causing them to gradually accumulate in the middle of the concave surface. Eventually, the lipid molecules bulge upward and combine with the positively charged amino acids on the concave surface to form a stable membrane curvature. How the “bending” process occurs is rarely mentioned in existing BAR–membrane interaction simulations. We briefly describe the role of the 4ATM BAR dimer in the bending process by starting from the existence of kinks, segmenting the BAR dimer, and analyzing the movement of lipid molecules. Our work provides feasible ideas for analyzing the role of BAR dimers in bending and reshaping lipid membranes. A deeper understanding of the role of BAR dimers will inform BAR protein engineering and BAR-based liposome application technologies. This research into BAR domains holds significant implications for understanding endocytosis and the intricate dynamics of the pathogen–host relationship. Also, by elucidating the BAR-mediated mechanisms of membrane bending and remodeling, we can gain insights into the fundamental processes that pathogens exploit to invade host cells. This knowledge is crucial for developing novel therapeutic strategies aiming at preventing and treating infections by targeting those critical molecular interactions. Moreover, a better understanding of BAR domain functions will advance the broader field of cellular transport. This offers potential applications in treating diseases linked to membrane dynamics, including neurological disorders and cancers. Thus, this study of BAR domains not only enhances our comprehension of cellular processes but also bridges the gap between basic science and clinical applications, underscoring its importance in both biomedical research and therapeutic development.

## Figures and Tables

**Table 1:** Simulation system setting.

System	Number	Time/ns	Size
4ATM + membrane	20	300	10.0 × 34.5 × 20.9 nm^3^
Membrane only	1	300	7.6 × 23.0 × 15.2 nm^3^

**Table 2:** Composition of the membrane.

Lipid Molecule	Proportion
Cholesterol	~32%
DOPC	~35%
DOPE	~17%
DOPS	~2%
PI (4,5) P_2_	~3%
Brain SM	~11%

**Table 3:** Major force-bearing residues. Red names indicate positively and negatively charged amino acids and green names indicate amino acids with benzene ring side chains.

Ranking	Helix 1–Helix 2	Helix 1–Helix 3	Helix 2–Helix 3
1	LYS 132	GLU 52	ARG 134
2	GLU 50	ARG 198	ARG 138
3	GLU 153	GLN 49	TYR 145
4	TRP 106	TRP 195	PHE 180
5	PHE 46	TYR 202	PHE 173
6	GLN 71	TYP 63	LEU 212
7	ARG 138	GLU 213	PHE 183
8	ARG 149	TYP 42	GLU 190
9	HIS 82	LEU 191	PHE 216
10	LEU 64	GLN 188	LEU 223

## Data Availability

The data supporting the findings of this article are available upon email request to the corresponding author.

## Supplementary Materials

The following supporting information can be downloaded at https://www.mdpi.com/article/10.3390/pathogens13100902/s1, Supplementary material with supporting information is available on the publisher’s website along with the published article. Figure S1: Simulation system trimming process; Figure S2: Force distribution analysis between Helix 1 and Helix 2; Figure S3: Force distribution analysis between Helix 1 and Helix 3; Figure S4: Force distribution analysis between Helix 2 and Helix 3; Figure S5: Pearson correlation coefficient between Arm segments (***α***), Core segments (***β***), Helix 2 (***γ***), and Span distance (***d***); Figure S6: Interaction area between Chain A and Chain B of the 4ATM BAR dimer.
