# Supplementary material for: Molecular Dynamics Reveal Key Steps in BAR-Related Membrane Remodeling
Source: Pathogens. 2024 Oct 15;13(10):902. doi: 10.3390/pathogens13100902 (PMC11510478; doi:10.3390/pathogens13100902)
Supplement: Supplementary file 1 [file pathogens-13-00902-s001.zip › pathogens-3215087-supplementary.pdf]

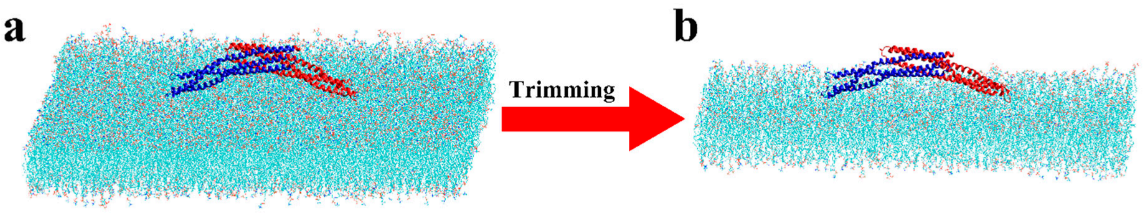

Figure S1. Simulation system trimming process. (a). 4ATM BAR dimer – membrane system before trimming. (b). 4ATM BAR dimer – membrane system after trimming.

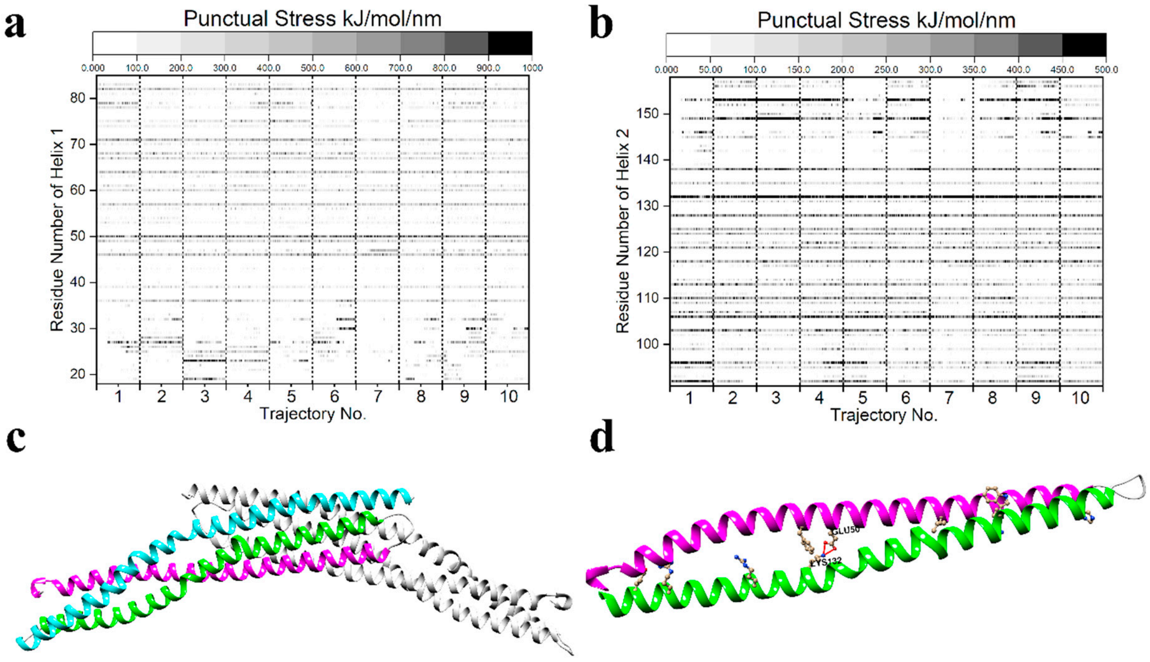

Figure S2. Force distribution analysis of the interactions between Helix 1 and Helix 2. Punctual stress represents the interaction force experienced by a residue on one helix from the other helix. (a) Punctual stress experienced by residues of Helix 1, averaged over 10 representative trajectories. (b) Punctual stress experienced by residues of Helix 2, averaged over 10 representative trajectories. (c) The three helices of the 4ATM BAR domain are color-coded: purple for Helix 1, green for Helix 2, and cyan for Helix 3. (d) Zoomed-in view of Helix 1 (purple) and Helix 2 (green), highlighting the top 10 residues with the highest punctual stress.

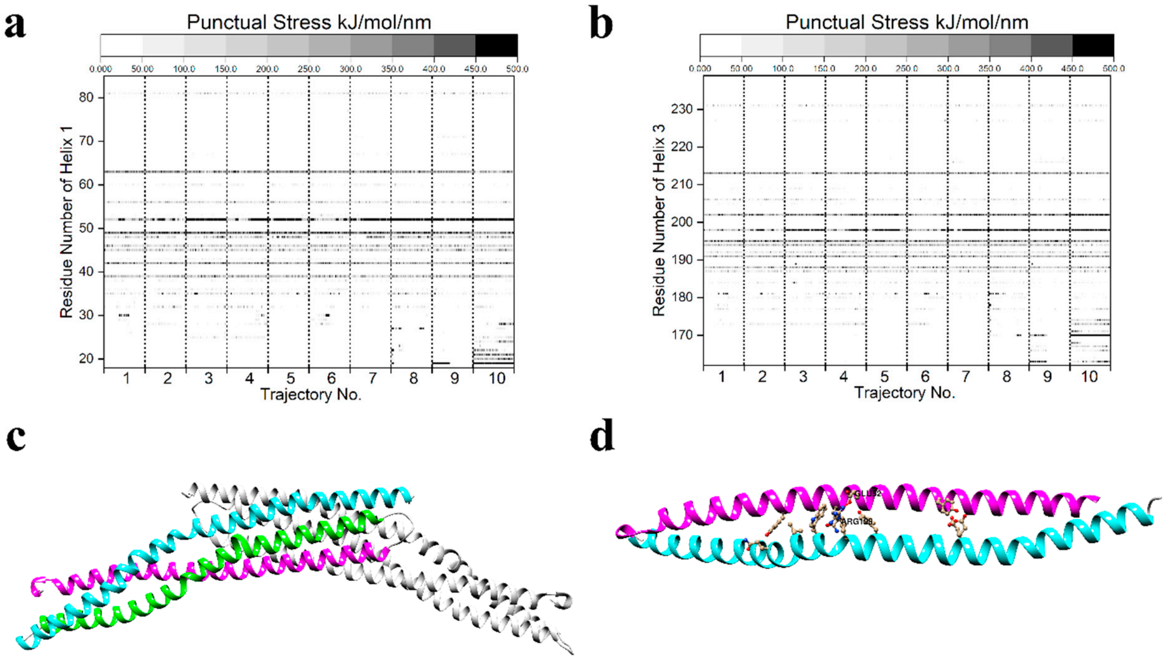

Figure S3. Force distribution analysis of the interaction between Helix 1 and Helix 3. Punctual stress denotes the interaction force experienced by a residue on one helix due to another helix. (a) Punctual stress on residues of Helix 1, based on 10 representative trajectories. (b) Punctual stress on residues of Helix 3, based on 10 representative trajectories. (c) The three helices of the 4ATM BAR domain are depicted in different colors: purple for Helix 1, green for Helix 2, and cyan for Helix 3. (d) A zoomed-in view of Helix 1 (purple) and Helix 3 (cyan), highlighting the top 10 residues with the highest punctual stress.

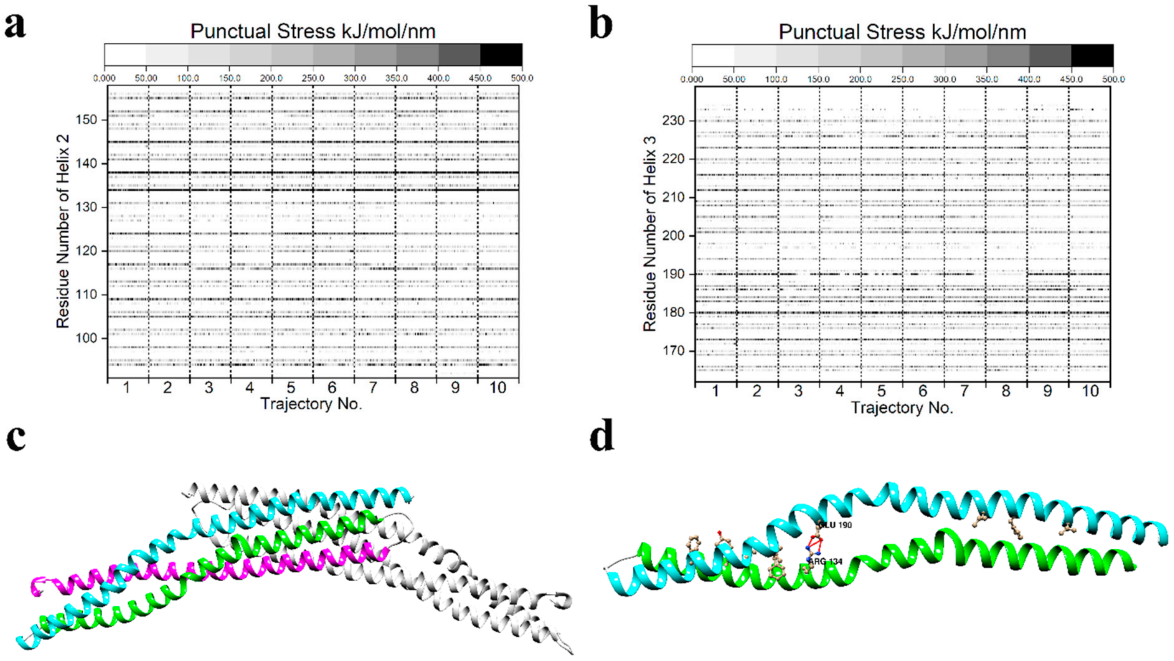

Figure S4. Force distribution analysis of the interaction between Helix 2 and Helix 3. Punctual stress represents the interaction force exerted on a residue of one helix by another helix. (a) Punctual stress on residues of Helix 2, based on 10 representative trajectories. (b) Punctual stress on residues of Helix 3, based on 10 representative trajectories. (c) The three helices of the 4ATM BAR domain are depicted in different colors: purple represents Helix 1, green represents Helix 2, and cyan represents Helix 3. (d) A zoomed-in view of Helix 2 (green) and Helix 3 (cyan), highlighting the top 10 residues with the highest punctual stress.

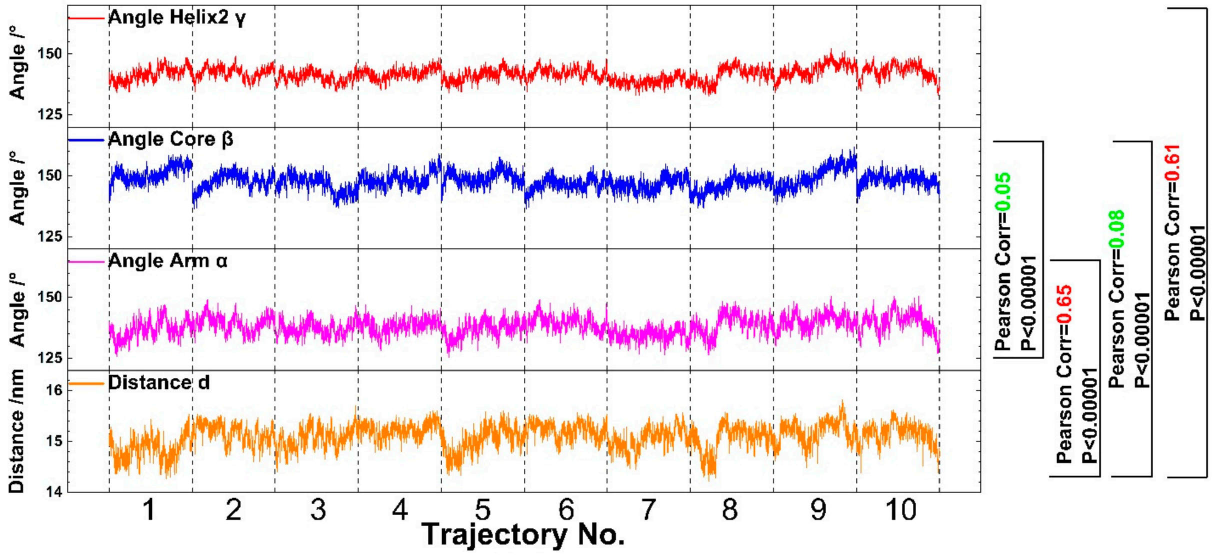

Figure S5. Pearson correlation coefficient between "arm segment" (α), "core segment" (β), Helix 2(γ), and span distance(d).

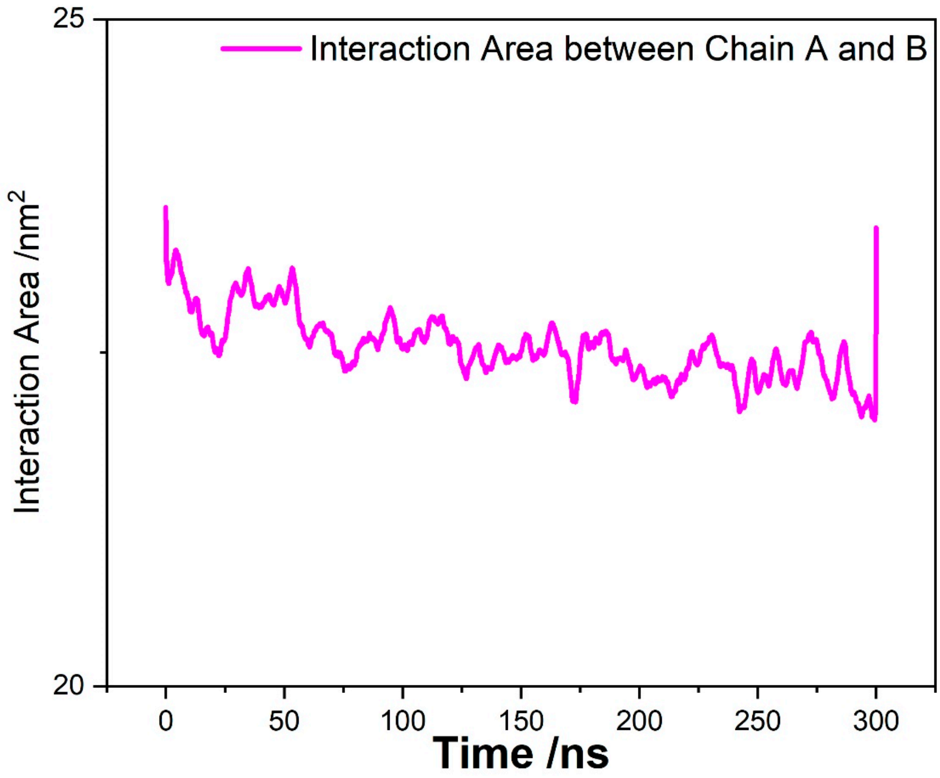

Figure S6. Interaction area between Chain A and Chain B of 4ATM BAR dimer.
